# mTOR-Controlled Autophagy Requires Intracellular Ca^2+^ Signaling

**DOI:** 10.1371/journal.pone.0061020

**Published:** 2013-04-02

**Authors:** Jean-Paul Decuypere, Dimphny Kindt, Tomas Luyten, Kirsten Welkenhuyzen, Ludwig Missiaen, Humbert De Smedt, Geert Bultynck, Jan B. Parys

**Affiliations:** Laboratory of Molecular and Cellular Signaling, Department of Cellular and Molecular Medicine, KU Leuven, Leuven, Belgium; Cornell University, United States of America

## Abstract

Autophagy is a lysosomal degradation pathway important for cellular homeostasis and survival. Inhibition of the mammalian target of rapamycin (mTOR) is the best known trigger for autophagy stimulation. In addition, intracellular Ca^2+^ regulates autophagy, but its exact role remains ambiguous. Here, we report that the mTOR inhibitor rapamycin, while enhancing autophagy, also remodeled the intracellular Ca^2+^-signaling machinery. These alterations include a) an increase in the endoplasmic-reticulum (ER) Ca^2+^-store content, b) a decrease in the ER Ca^2+^-leak rate, and c) an increased Ca^2+^ release through the inositol 1,4,5-trisphosphate receptors (IP_3_Rs), the main ER-resident Ca^2+^-release channels. Importantly, buffering cytosolic Ca^2+^ with BAPTA impeded rapamycin-induced autophagy. These results reveal intracellular Ca^2+^ signaling as a crucial component in the canonical mTOR-dependent autophagy pathway.

## Introduction

Macroautophagy (further referred to as “autophagy”) is a cellular degradation process characterized by the transfer of cellular material in double-membranous vesicles, termed autophagosomes, to the lysosomes. After fusion with lysosomes, the autophagosomal cargo becomes degraded. This intracellular cargo can consist of proteins, lipids or even entire organelles [1]. Basal levels of autophagy contribute to the maintenance of cellular homeostasis by removing damaged or toxic intrinsic components (*e.g.* damaged organelles, protein aggregates) [2]. Additionally, autophagy becomes stimulated during conditions of cellular stress. In these conditions, the recycling of their own material provides the cells with cellular building blocks that can be incorporated in newly synthesized macromolecules required for cellular anti-stress responses and energy production, so ensuring survival. Because of its role in these vital cellular functions, autophagy is implicated in various pathologies (reviewed in [3]).

The canonical signaling protein in autophagy regulation is the mammalian target of rapamycin (mTOR), a ubiquitous protein kinase that is also involved in the regulation of cell growth, proliferation, motility, protein translation and transcription [4]. Depending on its binding partners, mTOR forms two different protein complexes (mTORC1 and mTORC2), but only mTORC1 is directly involved in autophagy regulation. In growth-promoting conditions, active mTORC1 inhibits autophagy through phosphorylation of the unc-51-like kinase (ULK) 1/2 complex members. Upon certain stress conditions, mTORC1 becomes inhibited, alleviating these phosphorylations, and allowing the activation of the autophagic ULK1/2 complex [5]. In this way, inhibition of mTORC1 will activate autophagy in response to amino-acid depletion, growth-factor depletion, low energy production or chemical mTORC1 inhibitors, like rapamycin. Additionally, the activity of mTORC1 is regulated by its association/dissociation from the lysosomal membranes, mediated by Rag GTPase heterodimers [6].

Intracellular Ca^2+^ signaling was recently recognized as an important player in the regulation of autophagy, although its exact role still remains a matter of debate [7], [8]. On the one hand, Ca^2+^ signals mediated by the inositol 1,4,5-trisphosphate (IP_3_) receptor (IP_3_R), a ubiquitous endoplasmic-reticulum (ER) Ca^2+^-release channel, were reported to inhibit autophagy [9], [10], [11]. On the other hand, an increase in the cytosolic [Ca^2+^] enhanced autophagy [12], [13], [14], [15]. The exact role of Ca^2+^ and/or IP_3_Rs probably depends on the cellular state: in growth-promoting conditions constitutive IP_3_R-mediated Ca^2+^ signals from the ER to the mitochondria promote cellular bioenergetics and so inhibit basal autophagy, while during stress different, possibly cytosolic, Ca^2+^ signals stimulate autophagy [7].

The view that Ca^2+^ stimulates autophagy is based on several reports using different Ca^2+^-mobilizing compounds that stimulate autophagy [12], [13], [16], [17]. Recently, we observed that also starvation-induced autophagy was dependent on IP_3_R-mediated Ca^2+^ signaling [18]. Interestingly, starvation led to a sensitization of the intracellular Ca^2+^ machinery in different cell types, enhancing their Ca^2+^-signaling capacity. Moreover, the results suggested that this sensitization was operative in promoting autophagy-stimulating Ca^2+^ signals.

Since starvation not only acts on mTORC1, but can also affect a variety of cellular targets that may cause this sensitization, we now aimed to unravel the role of intracellular Ca^2+^ signaling in autophagy induced by rapamycin, a chemical compound that specifically inhibits mTORC1 [19]. Here, we found that, similar to starvation, rapamycin treatment increased the ER Ca^2+^-store content and resulted in more release through the IP_3_Rs. Moreover, intracellular Ca^2+^ signals were essential for rapamycin-induced autophagy. These findings identify intracellular Ca^2+^ signaling as a novel and essential component in the canonical mTOR-dependent autophagy pathway.

## Materials and Methods

### Cell culture

Doxycycline-inducible Atg5-knockout mouse embryonic fibroblasts (MEF cells), a kind gift from Prof. N. Mizushima (Tokyo Medical and Dental University, Japan), and wild-type human cervix carcinoma HeLa cells were grown in Dulbecco's modified Eagle's medium (DMEM) supplemented with 10% heat-inactivated fetal calf serum (FCS) and 10 mM HEPES buffer. The cells were grown at 37°C and 5% CO_2_ in the presence of 85 IU ml^−1^ penicillin and 85 µg ml^−1^ streptomycin. Knockdown of Atg5 in MEF was achieved by addition of 10 ng ml^−1^ doxycycline (Sigma-Aldrich NV, Diegem, Belgium) 2 days before the experiment [20]. Medium was changed regularly to avoid nutritional stress. All materials were purchased from Gibco, Life Technologies (Ghent, Belgium).

### Antibodies and reagents

The following antibodies were used for Western-blotting experiments: anti-GAPDH (G8795, Sigma-Aldrich NV), anti-BiP (G8918, Sigma-Aldrich NV), anti-LC3 (0231-100, NanoTools Antikörpertechnik GmbH & Co., Teningen, Germany), anti-SERCA2 (9580, Cell Signaling Technologies, Danvers, MA), anti-S6Rp and anti-phospho-S6Rp (8207, Cell Signaling Technologies), anti-Atg12 (2011, Cell Signaling Technologies) and anti-calreticulin (anti-CRT) (PA1-903, Thermo Fisher Scientific, Erembodegem, Belgium). The chemicals used were: A23187 and IP_3_ (Sigma-Aldrich NV), EGTA (Acros Organics BVBA, Geel, Belgium), thapsigargin (Enzo Life Sciences BVBA, Antwerp, Belgium), ionomycin, rapamycin and bafilomycin A1 (LC laboratories, Woburn, MA), ATP (Roche Diagnostics, Vilvoorde, Belgium), ^45^Ca^2+^ (PerkinElmer, Zaventem, Belgium), Fura2-AM (Biotium, Hayward, CA), and BAPTA-AM (Molecular Probes, Life Technologies).

### Fluorescent [Ca^2+^] measurements in intact cells

HeLa or MEF cells were seeded in 96-well plates (Greiner Bio-one BVBA, Wemmel, Belgium) at a density of approximately 1.2×10^4^ cells cm^−2^ and investigated 2 days after seeding. The cells were loaded with the ratiometric Ca^2+^ dye Fura2-AM (5 µM) for 30 min at 25 °C in modified Krebs solution containing 135 mM NaCl, 5.9 mM KCl, 1.2 mM MgCl_2_, 11.6 mM HEPES (pH 7.3), 11.5 mM glucose and 1.5 mM Ca^2+^. They were then incubated for at least 30 min in the absence of Fura2-AM. Fluorescence was monitored on a FlexStation-3 microplate reader (Molecular Devices, LLC, Sunnyvale, CA) by alternately exciting the Ca^2+^ indicator at 340 and 380 nm and measuring fluorescence emission at 510 nm.

### ^45^Ca^2+^ measurements in permeabilized cells

Unidirectional ^45^Ca^2+^-flux experiments were basically performed at 25°C as previously described [21], [22]. After permeabilization of HeLa cells with 20 µg ml^−1^ saponin, the non-mitochondrial Ca^2+^ stores were loaded for 45 min in 120 mM KCl, 30 mM imidazole (pH 6.8), 5 mM MgCl_2_, 5 mM ATP, 0.44 mM EGTA, 10 mM NaN_3_ and 150 nM free ^45^Ca^2+^ (28 µCi ml^−1^). Efflux medium containing 120 mM KCl, 30 mM imidazole (pH 6.8) and 1 mM EGTA was subsequently added and replaced every 2 min. IP_3_ (0.7 µM) was added during 2 min after 10 min of efflux. Eight min later, the ^45^Ca^2+^ remaining in the stores was released by incubation with sodium dodecyl sulfate during 30 min. The amount of ^45^Ca^2+^ present in each sample was measured using a Liquid Scintillation Analyzer (Packard BioScience, PerkinElmer).

### Calibration of the resting [Ca^2+^]

After trypsinization, suspensions of 5×10^6^ cells ml^−1^ of intact HeLa cells were loaded for 30 min with 5 µM Fura2-AM at 25°C in modified Krebs solution. The cells were then incubated for another 30 min in the absence of Fura2-AM. Fluorescence was monitored in the cell suspensions at 25°C in an AMINCO-Bowman Series 2 spectrofluorometer (Thermo Electron Corporation, Rochester, NY) by alternately exciting the Ca^2+^ indicator at 340 and 380 nm and recording emission fluorescence at 510 nm. After 50 s, 0.06 mg ml^−1^ digitonin was added to permeabilize the plasma membrane and to record fluorescence at a maximal [Ca^2+^]. Minimal fluorescence was measured 100 s later by adding 33 mM EGTA. The cytosolic [Ca^2+^] was derived using the following equation: *K-d.×Q×,(R-,R-min.)-(,R-max.-R)*. K_d_ is the dissociation constant of Fura2 for Ca^2+^ (241 nM), Q is the fluorescence ratio of the emission intensity excited by 380 nm in the absence of Ca^2+^ to that in the presence of saturating Ca^2+^, R is the fluorescence ratio, and R_min_ and R_max_ are the minimal and maximal fluorescence ratios, respectively.

### Immunoblots

HeLa or MEF cells were scraped into ice-cold phosphate-buffered saline and lysed in a modified RIPA buffer containing 10 mM sodium phosphate (pH 7.5), 150 mM NaCl, 1.5 mM MgCl_2_, 0.5 mM DTT, 1% Triton X-100, 10% glycerol and Complete EDTA-free Protease Inhibitor Tablets (Roche Diagnostics). After 30 min of incubation on ice, the lysates were cleared *via* centrifugation. Protein concentrations were determined by the Bradford procedure. For sample separation we used commercial Tris-Glycine or Bis-Tris SDS-PAGE gels (Invitrogen, Life Technologies). After transfer to a PVDF membrane (Immobilon®-P, Merck Millipore, Billerica, MA) the membranes were blocked with Tris-buffered saline containing 0.1% (v/v) Tween-20 and 5% (w/v) non-fat dry milk powder. Subsequently the membranes were incubated with primary antibody and horseradish peroxidase-conjugated secondary antibody. The immunoreactive bands were visualized with ECL substrate and exposed to CL-XPosure™ film (Thermo Fisher Scientific). The film was developed using a Kodak X-Omat 1000. Alternatively, alkaline phosphatase-conjugated secondary antibodies were used and visualized with a Storm 840 imager (GE Healthcare GmbH, Diegem, Belgium). Quantification was done with ImageJ software (rsbweb.nih.gov/ij/).

### GFP-LC3 measurements

HeLa cells were transfected with pcDNA3.1(-)-GFP-LC3 [18] with jetPRIME™ from Polyplus Transfection (Illkirch, France). 48 h later, the cells were fixated in 4% paraformaldehyde. Cells were then analyzed on a Zeiss LSM510 confocal microscope using a 63× lens with resolution near Nyquist rate (xy dimensions: ∼0.09 µm, z dimension: 0.14 µm). The number of punctae per cell was determined using an adapted version of the WatershedCounting3D plug-in for ImageJ [23], using a threshold for punctae volumes corresponding to autophagosome diameters of 0.5 µm. Only cells displaying a modest overexpression level were included in the analysis.

### Statistical analysis

Results are expressed as means±SEM, and *n* refers to the number of independent experiments. For statistical analyses, normal distribution (Shapiro-Wilk test) and equal variance (Levene's test) were first tested. Accordingly, significance was determined using the appropriate tests, as mentioned in the figure legends. Differences were considered significant at *p*<0.05.

## Results

### Rapamycin induces autophagy in a time- and concentration-dependent manner

**Figure 1:**
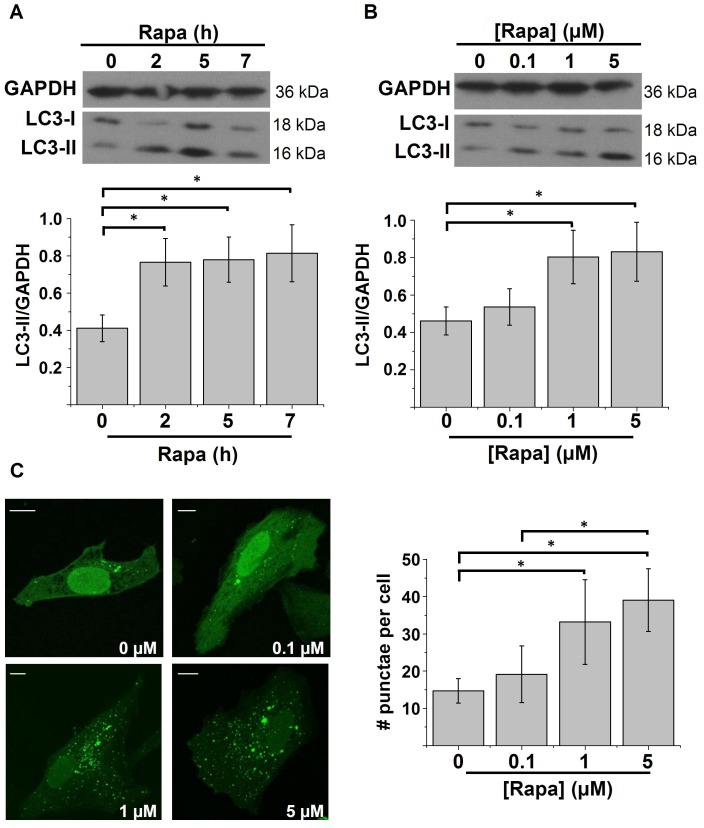
Time- and concentration-dependent stimulation of autophagy by rapamycin. A–B) Western-blot analysis for GAPDH and LC3 of protein lysates obtained from HeLa cells treated with DMSO or 1 µM rapamycin (Rapa) for the indicated time periods (A) (*n* = 7) or for 5 h with the indicated concentrations (B) (*n* = 6). One hour before harvesting, 100 nM bafilomycin A1 was added. Upper panels: representative Western blots; lower panels: quantification of the LC3-II/GAPDH ratio. C) GFP-LC3-punctae quantification in HeLa cells treated for 5 h with different concentrations of rapamycin. Left: representative pictures. The scale bar represents 10 µm. Concentrations are mentioned in the right lower corner. Right: Quantification of the number of punctae per cell (*n* = 3). * *p*<0.05, repeated measurements ANOVA.

We treated HeLa cells with 1 µM of rapamycin for different time periods (2, 5 and 7 h) or for 5 h with different concentrations of rapamycin (0.1, 1 and 5 µM). First, the inhibition of mTORC1 by rapamycin was verified by assessing the phosphorylation of one of the downstream targets of mTORC1, S6 ribosomal protein (S6Rp), using a phospho-specific S6Rp antibody [24]. In all treatment conditions using rapamycin, the phosphorylation of S6Rp was inhibited (Fig. S1). Subsequently, autophagy was assessed by immunoblotting for detection of the essential autophagy protein LC3. In the autophagic pathway, this protein is conjugated to phosphatidylethanolamine and thereby recruited to the autophagosomal membrane. This lipidated form of LC3 can be detected as a band with an apparently lower molecular weight (LC3-II, 16 kDa) than the non-lipidated, non-autophagic form (LC3-I, 18 kDa). The level of LC3-II is therefore an indication for the extent of autophagy [25]. However, since LC3-II remains associated with the autophagosomes, it eventually becomes degraded in the lysosomes. Therefore, increased LC3-II levels can also be explained by defective autophagic flux and hence accumulation of LC3-II-positive autophagosomes. The addition of lysosomal inhibitors (*e.g.* bafilomycin A1) is therefore recommended as a proper control condition to verify ‘truly’ increased autophagy induction [26], [27]. Therefore, bafilomycin A1 (100 nM) was added during the last hour of our treatment and the formation of LC3-II was monitored in this last hour (quantified as the LC3-II/GAPDH ratio, as recommended [27]). Our results show that LC3-II levels were increased consequently to both increasing time periods and concentrations of rapamycin treatment (Fig. 1A–B).

We also tested the effect of different concentrations of rapamycin on the localization of transiently expressed GFP-LC3 in HeLa cells. Autophagic GFP-LC3-II will concentrate at the autophagosomes, which can be detected as intracellular GFP-LC3 punctae. The amount of these punctae per cell correlates with the level of autophagy [27]. The number of GFP-LC3 punctae per cell was significantly increased upon rapamycin treatment (Fig. 1C). In agreement with the results obtained by LC3 Western blotting, the lowest concentration of rapamycin (0.1 µM) did not significantly increase the number of punctae.

### Rapamycin treatment increases the intracellular Ca^2+^-store content and IP_3_-induced Ca^2+^ release

**Figure 2:**
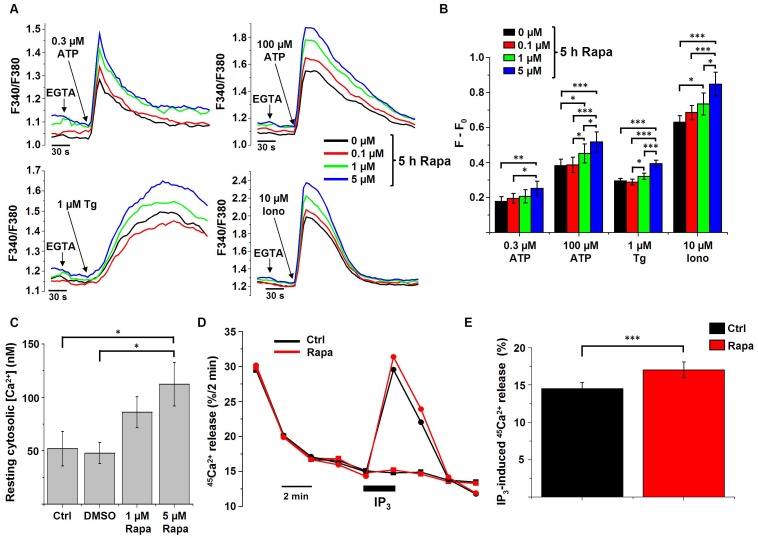
Rapamycin affects intracellular Ca^2+^ signaling. A) Representative measurements (*n* = 4) of cytosolic Ca^2+^ signals, displayed as Fura2 ratio (F340/F380), showing the effect of 0.3 µM and 100 µM ATP, 1 µM thapsigargin (Tg) or 10 µM ionomycin (Iono) in intact HeLa cells treated with different concentrations of rapamycin (Rapa) for 5 h. 45 s prior to the addition of ATP, Tg or Iono, EGTA (3 mM) was given to buffer extracellular Ca^2+^ as indicated. B) Quantification of the average amplitude of the response (F−F_0_) (*n* = 4). * *p*<0.05; ** *p*<0.01; *** *p*<0.001, repeated measurements ANOVA. C) Mean resting cytosolic [Ca^2+^], measured in Fura2-loaded HeLa cells treated with the indicated concentrations of rapamycin for 5 h, as well as in the absence (Ctrl) or presence of DMSO (*n* = 3). * *p*<0.05, repeated measurements ANOVA. D) Unidirectional ^45^Ca^2+^-flux experiments in permeabilized cells pretreated with 1 µM rapamycin for 5 h or with DMSO (Ctrl). Mean fractional ^45^Ca^2+^ release (%/2 min) is shown as a function of time with the effect of 0.7 µM IP_3_ (circles) or no addition (squares). The horizontal bar indicates the presence of IP_3_. E) Quantitative analysis of the IP_3_-induced ^45^Ca^2+^ release in cells pretreated for 5 h with 1 µM rapamycin or DMSO (Ctrl) (*n* = 8). *** *p*<0.001, paired Student's *t-*test.

We loaded HeLa cells, treated with or without rapamycin, with the fluorescent cytosolic Ca^2+^ dye Fura2 and measured the response upon addition of the Ca^2+^-ionophore ionomycin, thapsigargin or ATP. Ionomycin can be used to determine the size of all Ca^2+^ stores. Thapsigargin is an inhibitor of the SERCA pumps and can be used to determine the ER Ca^2+^ content. ATP binds to its receptor at the plasma membrane, resulting in the production of IP_3_ and consequently inducing IP_3_R-mediated Ca^2+^ release. Before treatment with the Ca^2+^-mobilizing agents, extracellular Ca^2+^ was chelated using 3 mM EGTA. As shown in Fig. 2A–B, cells treated with rapamycin concentrations triggering autophagy (1 and 5 µM) displayed an increased Ca^2+^ release in response to the different Ca^2+^-mobilizing agents tested. Interestingly, the lowest concentration (0.1 µM) of rapamycin did not result in a significantly increased Ca^2+^ release (Fig. 2B), correlating with its inability to significantly stimulate autophagy.

The traces from Fig. 2A before EGTA addition also suggest an increase in the resting cytosolic [Ca^2+^] upon rapamycin treatment. To verify this behavior, the Fura2-ratio signal was calibrated, revealing a significant increase in the cytosolic [Ca^2+^] in cells treated with rapamycin (Fig. 2C). As a control, it was verified that rapamycin addition by itself did not induce a shift in the spectral characteristics of the Fura2 signal (Fig. S2).

The results obtained with Fura2-loaded cells point to an increase in IP_3_R-mediated Ca^2+^ release after rapamycin treatment. To verify this hypothesis, we performed Ca^2+^-flux experiments in plasma membrane-permeabilized cells. The benefit of using plasma membrane-permeabilized cells is the direct access to the cytosol and the possibility to directly activate the IP_3_R *via* the addition of IP_3_. In this way, the extent of the IP_3_R-mediated Ca^2+^ release can be assessed in a quantitative way without interference of plasma-membrane Ca^2+^ fluxes. The non-mitochondrial Ca^2+^ stores were loaded with ^45^Ca^2+^ to steady state and the release of ^45^Ca^2+^ from the cell layer was then measured every 2 min. We added IP_3_ at a submaximal concentration (0.7 µM) and measured IP_3_-induced Ca^2+^ release (Fig. 2D). In these conditions, IP_3_-induced Ca^2+^ release was enhanced in cells treated with 1 µM rapamycin for 5 h (Fig. 2E).

Thus, these independent Ca^2+^ assays indicate that an optimization of the Ca^2+^ signaling occurs upon rapamycin treatment by increasing the ER Ca^2+^-store content and the IP_3_R-mediated Ca^2+^ release.

### Rapamycin treatment reduces the ER Ca^2+^-leak rate

**Figure 3:**
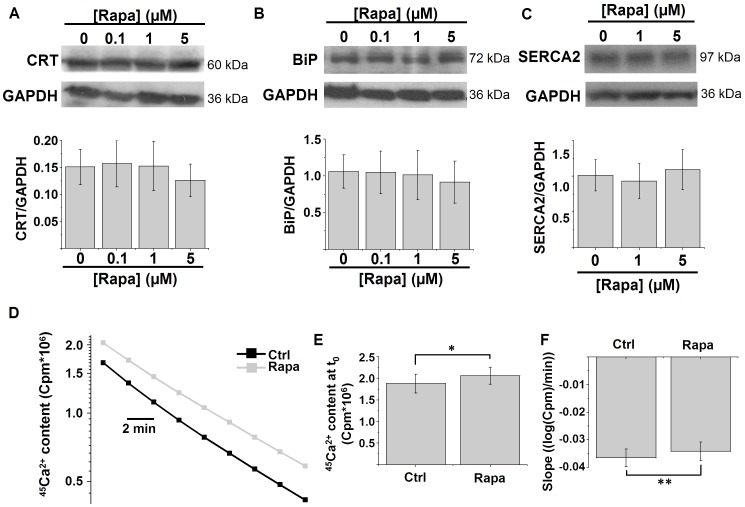
Rapamycin reduces the ER Ca^2+^-leak rate. A–B) Western-blot analysis for luminal Ca^2+^-binding proteins in HeLa cells treated with the indicated concentrations of rapamycin (Rapa) for 5 h: calreticulin (CRT) (A) and BiP/Grp78 (BiP) (B). Upper panels: representative Western blots; lower panels: quantification of the protein/GAPDH ratio (*n* = 4). C) Western-blot analysis for SERCA2 in HeLa cells treated with the indicated concentrations of rapamycin for 5 h. Upper panels: representative Western blots; lower panel: quantification of the SERCA2/GAPDH ratio (*n* = 4). D) Representative plot showing the decrease in ER ^45^Ca^2+^ content (logarithmic scale) in a Ca^2+^-free efflux medium without ATP as a function of time in permeabilized HeLa cells pretreated for 5 h with 1 µM rapamycin or with DMSO. The passively bound Ca^2+^ was determined by loading the cells with ^45^Ca^2+^ in the presence of 10 µM of the Ca^2+^ ionophore A23187 and then subtracted from the stored ^45^Ca^2+^. The ER Ca^2+^-leak rate can be estimated as the rate of decline of the ER ^45^Ca^2+^-store content as a function of time. E) Quantification of the mean ^45^Ca^2+^-store content at the beginning of the measurement (t_0_) (*n* = 5). F) Quantification of the mean slope of the curve in D after transformation to a linear scale, which is a measure of the ^45^Ca^2+^-leak rate (*n* = 5). * *p*<0.05; ** *p*<0.01, paired Student's *t*-test.

To evaluate the underlying cause of the increased ER Ca^2+^-store content upon rapamycin treatment, we analyzed several parameters that control the ER Ca^2+^ content. First, we analyzed the main Ca^2+^-buffering proteins of the ER: calreticulin and BiP/Grp78. Rapamycin treatment, however, did not significantly affect the levels of these proteins (Fig. 3A–B). We also assessed the levels of SERCA2, the major Ca^2+^-pump isoform in the ER of HeLa cells, but rapamycin treatment did not alter SERCA2 levels (Fig. 3C).

Finally, we also measured the Ca^2+^-leak rate using ^45^Ca^2+^-flux experiments in permeabilized cells, as previously described [28]. Cells were loaded with ^45^Ca^2+^ in the absence or in the presence of the Ca^2+^ ionophore A23187, the latter to determine the passively bound Ca^2+^. The value for the passively bound Ca^2+^ is then subtracted to calculate exclusively the amount of releasable Ca^2+^ in the internal stores. This experiment also revealed a significantly increased Ca^2+^-store content (Fig. 3D and Fig. 3E), similarly to the findings in the intact Fura2-loaded cells (Fig. 2A–B). The ER Ca^2+^-leak rate can be appreciated by the slope of the curve plotting the Ca^2+^ content that remains in the cell layer as (logarithmic scale) a function of time. As shown in Fig. 3D and the quantification in Fig. 3F, rapamycin treatment slightly but significantly reduced the slope of the curve and hence the Ca^2+^-leak rate.

In conclusion, rapamycin treatment reduced the ER Ca^2+^-leak rate, which may account for the increased Ca^2+^-store content observed.

### Rapamycin-induced changes in Ca^2+^ signaling are independent of functional autophagy and occur upstream of the Atg12-Atg5 complex

**Figure 4:**
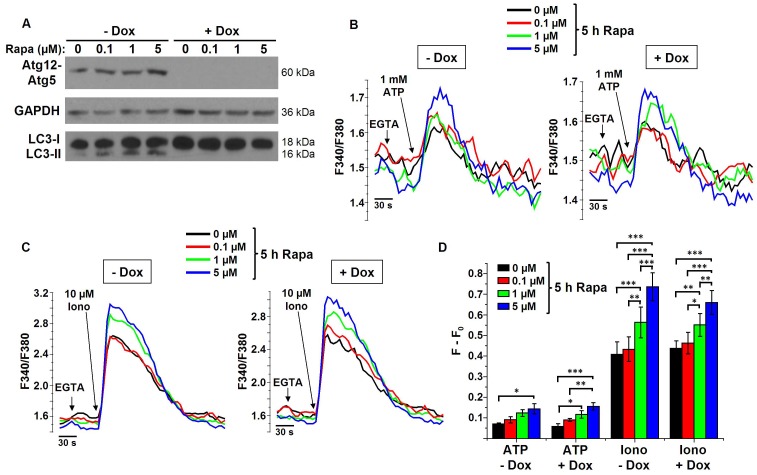
Changes in Ca^2+^ signaling are independent of autophagy stimulation and occur upstream of the Atg12-Atg5 complex. A) Representative Western-blot analysis for Atg12 (showing the autophagic Atg12-Atg5 complex), GAPDH and LC3 of protein lysates obtained from MEF cells pretreated with (+Dox) or without (-Dox) doxycycline and treated with DMSO or 0.1, 1 or 5 µM rapamycin (Rapa) for 5 h (*n* = 3). B–C) Representative measurements of cytosolic Ca^2+^ signals, displayed as Fura2 ratio (F340/F380), showing the effect of 1 mM ATP (B) or 10 µM ionomycin (Iono) in intact MEF cells pretreated with or without doxycycline and treated with different concentrations of rapamycin for 5 h. Prior to the addition of ATP or Iono, EGTA (3 mM) was added to chelate the extracellular Ca^2+^ as indicated. D) Quantification of the average amplitude of the response (F−F_0_) (*n* = 3, 4, 5 and 6 for ATP-Dox, ATP+Dox, Iono-Dox and Iono+Dox, resp.) * *p*<0.05; ** *p*<0.01; *** *p*<0.001, repeated measurements ANOVA.

To analyze whether the observed changes in Ca^2+^ signaling during rapamycin treatment are upstream or downstream of autophagy stimulation, we performed [Ca^2+^] measurements in doxycycline-inducible Atg5-knockout MEF cells. The addition of doxycycline to the medium results in the complete knockdown of Atg5, the absence of the autophagic Atg12-Atg5 complex and the inability to stimulate autophagy by rapamycin (Fig. 4A) [20]. [Ca^2+^] measurements in MEF cells showed a similar increase in the ATP- and ionomycin-induced Ca^2+^ release upon rapamycin treatment as in HeLa cells (Fig. 4B–D), indicating that these effects do not depend on the cell type. Even more interestingly, in the absence of Atg5, similar changes in Ca^2+^ signaling were observed, indicating that the rapamycin-induced increase in Ca^2+^ signaling is independent of functional autophagy.

### Intracellular Ca^2+^ is required for rapamycin-induced autophagy

**Figure 5:**
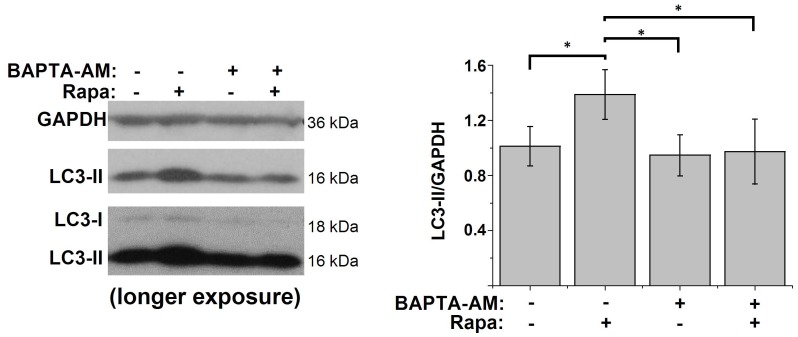
Rapamycin-induced autophagy is Ca^2+^-dependent. Western-blot analysis for GAPDH and LC3 of protein lysates obtained from HeLa cells treated for 5 h with DMSO, 1 µM rapamycin (Rapa), 10 µM BAPTA-AM or both. One hour before harvesting, 100 nM bafilomycin A1 was added. Left: representative Western blots; right: quantification of the LC3-II/GAPDH ratio (*n* = 6). * *p*<0.05, repeated measurements ANOVA.

Since we observed changes in the Ca^2+^ machinery by rapamycin treatment that correlated with the induction of autophagy, we investigated whether intracellular Ca^2+^ signals played a role in rapamycin-induced autophagy. Therefore, we incubated HeLa cells during the rapamycin treatment (1 µM, 5 h) with the intracellular Ca^2+^ chelator BAPTA-AM (10 µM). Although incubation with BAPTA-AM had no significant effect on the basal levels of autophagy, rapamycin-induced autophagy was abolished by loading the cells with BAPTA-AM (Fig. 5). These results indicate that cytosolic Ca^2+^ was required for rapamycin-induced autophagy.

## Discussion

The major finding of this study is the occurrence of changes in the intracellular Ca^2+^ homeostasis during rapamycin treatment that correlated with the stimulation of autophagy. These changes include an increase in the intracellular Ca^2+^-store content, a decrease in the ER Ca^2+^-leak rate and more IP_3_-induced Ca^2+^ release. This study also reveals that cytosolic Ca^2+^ is required for rapamycin-induced autophagy. These findings therefore identify intracellular Ca^2+^ as a novel and essential secondary messenger in the canonical mTOR-dependent autophagy pathway.

Recently, we have identified enhanced IP_3_R-mediated Ca^2+^ signaling as an essential player in starvation-induced autophagy [18]. We observed a sensitization of the cellular Ca^2+^-release machinery during starvation, leading to increased IP_3_R-mediated Ca^2+^ signaling from the ER Ca^2+^ stores. However, it was not clear whether the observed starvation-induced alterations in Ca^2+^ homeostasis were caused by mTORC1 inhibition, or by another pathway affected by starvation. In the present study, we therefore used rapamycin as a specific tool to chemically and irreversibly inhibit mTORC1. Our results now provide unequivocal evidence that mTORC1-dependent autophagy stimulation causes sensitization of Ca^2+^-signaling events and that these Ca^2+^ signals are essential to drive autophagy induced by mTORC1 inhibition. This is an important finding, since mTORC1 is the canonical upstream regulator of the autophagy pathway.

Similar to the effects of starvation, we found an increase in the ER Ca^2+^-store content during rapamycin treatment, leading to increased IP_3_-induced Ca^2+^ release. During starvation, the increase in the Ca^2+^-store content was associated with an increase in the levels of intraluminal Ca^2+^-buffering proteins and with a reduction in the ER Ca^2+^-leak rate [18]. During rapamycin treatment, the levels of the intraluminal Ca^2+^-buffering proteins remained unaltered, while the Ca^2+^-leak rate was clearly reduced. The unaltered levels of the Ca^2+^-buffering proteins suggest that they take no part in the regulation of the Ca^2+^-leak rate during rapamycin-induced autophagy, in contrast to the situation upon starvation [18], [29]. How the ER Ca^2+^ leak is regulated and which proteins are involved are however still a matter of debate [30].

In addition to the increased Ca^2+^-store content, we also observed increased IP_3_-mediated Ca^2+^ release after rapamycin treatment. However, in contrast to our findings, other reports revealed a decrease in the IP_3_R-mediated Ca^2+^ release after rapamycin treatment, which was due to decreased interactions of mTORC1-protein members with the IP_3_R, and subsequent less mTORC1-dependent IP_3_R phosphorylation [31], [32]. The reason for this discrepancy probably reflects experimental differences, including the time of rapamycin treatment (5–15 min in [32] *versus* 2–7 h in present study). Fifteen minutes of rapamycin treatment is probably not sufficient to cause autophagy stimulation and these short time periods were therefore not investigated in our study. In any case, the relevance of the mTORC1-dependent phosphorylation of the IP_3_R and its potential effect on IP_3_R activity after prolonged exposure to rapamycin requires further investigation. In addition, it should be noted that IP_3_Rs are also proposed to inhibit autophagy through two distinct mechanisms: as a Ca^2+^ channel [11] or as a scaffold protein [33]. In the former, IP_3_Rs inhibit autophagy through basal constitutive Ca^2+^ signaling towards mitochondria to fuel mitochondrial bioenergetics, thereby promoting ATP production and suppressing AMP-activated kinase AMPK [11]. In the latter model, IP_3_Rs promote the anti-autophagic interaction between Bcl-2 and Beclin 1 in a Ca^2+^-independent manner [33]. We recently pointed out that the exact role of IP_3_Rs in autophagy regulation is probably dependent on the cellular context, being different in basal *versus* stressed conditions [7].

In HeLa cells, we also detected an increase in the resting cytosolic [Ca^2+^] upon rapamycin treatment. The reason for this observation is unclear, and could possibly involve an enhanced Ca^2+^ influx across the plasma membrane. In contrast, MEF cells rather showed a reduced cytosolic [Ca^2+^] upon rapamycin treatment (Fig. 4), suggesting that the increase in the cytosolic [Ca^2+^] may be cell-type dependent, in contrast to the increase of the ER Ca^2+^-store content and agonist-induced Ca^2+^ release, which occurs in both cell types.

Finally, we also found that mTORC1-controlled autophagy was dependent on proper intracellular Ca^2+^ signaling, since chelating cytosolic Ca^2+^ by BAPTA-AM treatment completely abolished rapamycin-induced autophagy. In contrast, inhibiting autophagy by Atg5 knockout in MEF cells did not alter the observed rapamycin-induced changes in Ca^2+^ signaling. Taken together, these results suggest that the changes in Ca^2+^ signaling during rapamycin-induced autophagy are upstream of the Atg12-Atg5 complex and therefore identify intracellular Ca^2+^ as a novel critical player in the canonical mTORC1-dependent autophagy pathway.

The finding that intracellular Ca^2+^ is required for autophagy induction is in line with a series of reports showing that an increase in cytosolic [Ca^2+^] can stimulate autophagy [12], [13], [14], [15], [16], [17], [34]. Other reports however have assigned an inhibitory role for Ca^2+^ in autophagy regulation [10], [11], [35], [36]. We believe that this discrepancy may be explained by the specific role of different Ca^2+^ signals: a Ca^2+^ signal in normal growth-promoting conditions (probably targeted towards mitochondria) that inhibits basal autophagy and a different Ca^2+^ signal in conditions of cellular stress that stimulates autophagy (reviewed in [7]). We speculate that in order to generate these autophagy-stimulating Ca^2+^ signals, a sensitization of the Ca^2+^ machinery is required, as observed during starvation or during rapamycin treatment.

The target of this autophagy-stimulating Ca^2+^ signal remains elusive. CaMKKβ [12], [34], CaMKI [37], but also ERK [13] and PKCθ [16] have been proposed as potential targets for these cytosolic Ca^2+^ signals. As the exact target might depend on the stimulus or the cell type used, it is also likely that different downstream targets or pathways may be involved in the Ca^2+^-dependent regulation of autophagy.

In conclusion, intracellular Ca^2+^ signaling should be considered as an essential component of the canonical mTORC1-regulated autophagy pathway. The further characterization of this Ca^2+^-dependent pathway may reveal novel important players and targets in autophagy. Finally, affecting these intracellular Ca^2+^ signals by chemical compounds or genetic interventions may provide a unique way to modulate the canonical mTORC1-controlled autophagy pathway.

## Supporting Information

Figure S1 **Rapamycin inhibits S6Rp phosphorylation.** Western-blot analysis for total and phosphorylated S6Rp in HeLa cells treated with the indicated concentrations of rapamycin (Rapa) for 5 h or with 1 µM rapamycin for the indicated times. A representative blot is shown for 2 independent experiments.
(TIFF)

Figure S2 **Rapamycin addition does not induce a shift in the spectral characteristics of Fura2.** Representative measurements (*n* = 2) of cytosolic Ca^2+^ signals, displayed as Fura2 ratio (F340/F380), showing the effect of the acute addition of DMSO or different concentrations of rapamycin in intact HeLa cells; control denotes no addition. The arrow indicates the time of addition.
(TIFF)
