# Supplementary figures and images for: mTOR-Controlled Autophagy Requires Intracellular Ca2+ Signaling
Source: PLoS One. 2013 Apr 2;8(4):e61020. doi: 10.1371/journal.pone.0061020 (PMC3614970; doi:10.1371/journal.pone.0061020)

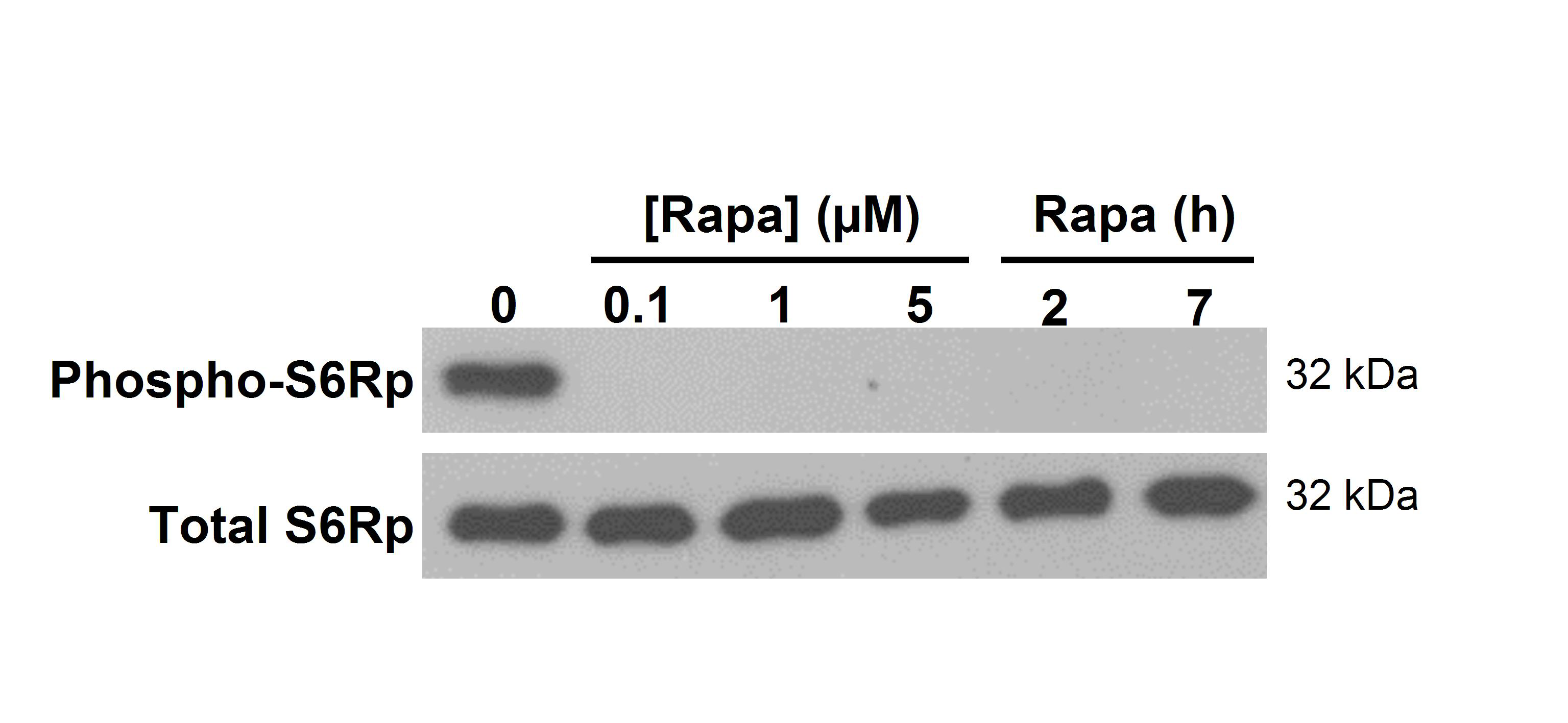

Supplement: Figure S1 — Rapamycin inhibits S6Rp phosphorylation. Western-blot analysis for total and phosphorylated S6Rp in HeLa cells treated with the indicated concentrations of rapamycin (Rapa) for 5 h or with 1 µM rapamycin for the indicated times. A representative blot is shown for 2 independent experiments. (TIFF) [file pone.0061020.s001.tif]

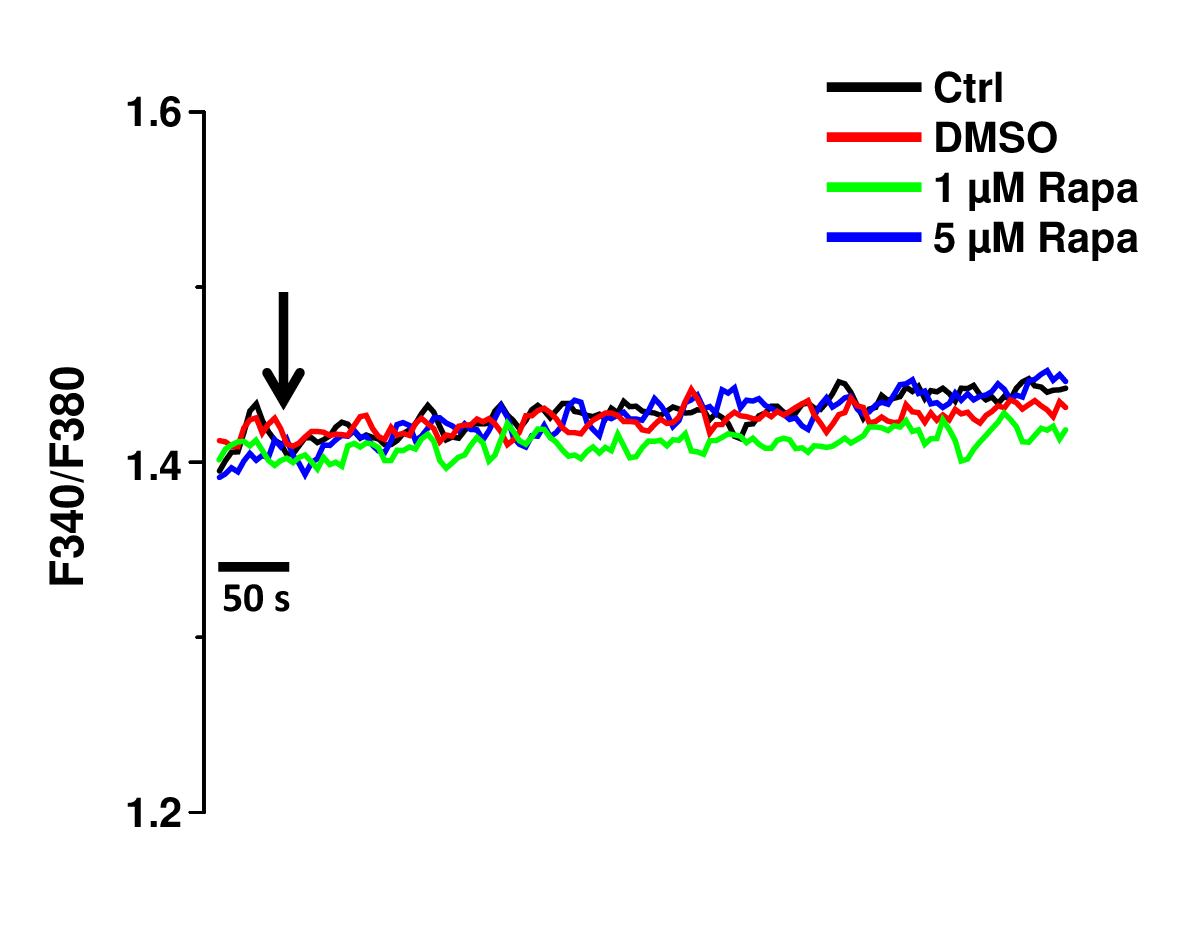

Supplement: Figure S2 — Rapamycin addition does not induce a shift in the spectral characteristics of Fura2. Representative measurements (n = 2) of cytosolic Ca2+ signals, displayed as Fura2 ratio (F340/F380), showing the effect of the acute addition of DMSO or different concentrations of rapamycin in intact HeLa cells; control denotes no addition. The arrow indicates the time of addition. (TIFF) [file pone.0061020.s002.tif]
